# Effects of Chemical Composition and Controlled Cooling on the Phase Transition in Coconut Oil Bio-Phase Change Materials

**DOI:** 10.1021/acsomega.6c01295

**Published:** 2026-07-02

**Authors:** Matheus N. Guedes, Erislene S. Almeida, Christianne E. C. Rodrigues, Roberta F. Neumeister, Taygoara F. Oliveira, Antonio C. P. Brasil, Simone Monteiro

**Affiliations:** † Laboratory of Energy and Environment, University of Brasília, Federal District, Brasilia 70910-900, Brazil; ‡ Department of Food Engineering, Separation Engineering Laboratory, University of São Paulo (USP), Pirassununga, São Paulo 13635-900, Brazil

## Abstract

Vegetable-oil-based phase change materials (bio-PCMs) are attractive for thermal energy storage due to their renewability, low cost, and high latent heat. However, their adoption is hindered by polymorphism, the ability of triacylglycerol molecules to form multiple crystalline arrangements during solidification. Each polymorph exhibits distinct melting ranges, enthalpies, and metastability, making the prediction of thermophysical properties challenging for the energy-materials community, which often treats PCMs as single-transition systems. This work demonstrates how chemical composition and polymorphic state fundamentally govern the phase transition behavior of coconut oil, using a combination of controlled T-history experiments and composition-based thermophysical modeling. A modified T-history apparatus with precise control over heating and cooling rates was developed, enabling the quantification of subcooling and assessment of how thermal history influences the material’s crystallization behavior. After validation with *n*-eicosane, the system was applied to chemically characterized coconut oil samples, and the resulting thermal responses were interpreted considering thermodynamic predictions for the enthalpies associated with the different polymorphic states. Coconut oil was selected as a model lipid system given the extensive compositional information available in the literature, allowing the mechanistic insights obtained here to be extrapolated to other vegetable oils when chemical characterization is available. By integrating fatty-acid and triacylglycerol profiles with T-history data, we show that (i) coconut oil exhibits subcooling levels that depend on both cooling rate and composition, though remaining limited (≤1 °C); and (ii) controlling crystallization conditions influences the predominance of specific polymorphs, enabling effective tuning of the transition temperature range and latent heat. Coconut oil also displayed stable thermophysical behavior over repeated heating/cooling cycles (<5% deviation), confirming its thermal stability. Overall, this study clarifies a key knowledge gap by demonstrating why vegetable oils cannot be treated as simple single-transition PCMs and how their phase change behavior can be rationally controlled. These insights establish a pathway for engineering reliable lipid-based PCMs through compositional and polymorphic design.

## Graphical abstract

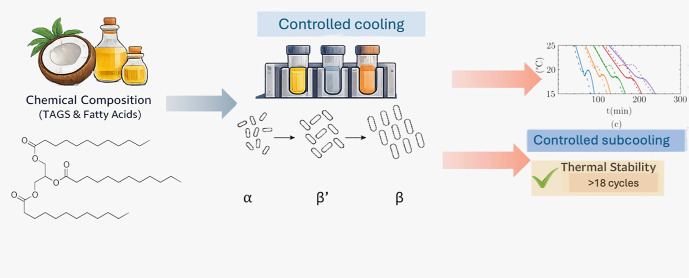

## 1 Introduction

Thermal energy storage (TES) has emerged as an essential strategy to reduce greenhouse gas emissions and alleviate global energy demands.^1–3^ In TES technologies, energy can be stored as sensible heat (related to temperature variation) or as latent heat (related to phase transitions). Among these alternatives, latent heat storage using phase change materials (PCM) is particularly attractive due to its high energy storage density and near-isothermal operation during charging and discharging.^4^ For indoor climatization alternatives, organic paraffins (linear alkanes) are widely used PCMs due to their predictable crystallization behavior and tunable melting temperatures. These materials are categorized according to carbon-chain length: compounds with 1–4 carbons are gaseous, those with 5–17 carbons are liquid paraffins, and chains longer than 17 carbons form solid waxes at room temperature. Their thermophysical properties depend strongly on molecular size, with longer carbon chains exhibiting higher melting points and latent heats of fusion. Typical values reported for paraffins include melting temperatures in the 30–90 °C range, latent heats of 180–230 J·g^–1^, and specific heat capacities around 2.1 kJ·kg^–1^·K^–1^, which explains their widespread adoption in low- and medium-temperature TES applications.^5^ The sharp transition temperature, latent heat, and specific heat capacity of commercial paraffins result from industrial fractionation processes, which allow their melting temperature and enthalpy to be tailored to specific applications, a level of control that motivates similar strategies for biobased PCMs.^6^ Nevertheless, growing environmental concerns and inherent flammability have motivated the search for sustainable and safer biobased alternatives.^7,8^

Among multiple biobased candidates, vegetable oils emerge as promising alternatives. They are abundant, renewable, and can be sourced from different species year-round.^8^ Furthermore, the valorization of waste oils from industrial processes presents opportunities for circular economy practices in the energy sector.^9^ Chemically, vegetable oils are complex mixtures of triacylglycerols (TAGs), whose composition and consequently, melting behavior, varies with plant source and growth conditions, typically exhibiting phase transitions in the −30 to 40 °C range.^10–12^ Different oils (also referred to as fats when in the solid state) such as fat waste from slaughterhouses,^13^ fats from confectionery industry,^14^ waste coconut and palm oils,^9^ coconut oil^15,16^ have already shown their potential as biobased PCMs.

The primary challenge and opportunity in utilizing vegetable oils as PCMs lies in their complex crystallization behavior. Despite extensive knowledge of TAG polymorphism in food and lipid sciences, its implications for designing reliable biobased PCMs remain largely underexplored. TAGs can solidify into multiple crystalline structures with distinct stabilities, melting points, and enthalpies, a phenomenon called polymorphism. This polymorphic tendency, influenced by chemical composition, thermal history, and crystallization kinetics, has often been viewed as a drawback for PCM applications due to the potential for irreproducible thermal performance. In lipid systems, crystallization is a kinetically controlled process in which cooling rate, crystallization temperature, and molecular organization govern nucleation and crystal growth pathways. As a result, the same material may exhibit different thermal responses under different processing conditions, not due to intrinsic instability, but due to variations in polymorphic selection and crystallization dynamics.^17,18^ However, a fundamental understanding of this behavior is key to engineering vegetable oil-based PCMs with tailored and reliable thermal properties. By controlling polymorphism, it may be possible to design materials that undergo predictable and stable phase transitions over countless thermal cycles. Accurately characterizing this complex phase behavior is crucial. However, most studies investigating vegetable oils as PCMs rely almost exclusively on Differential Scanning Calorimetry (DSC) measurements, which do not capture the bulk crystallization behavior relevant to practical TES applications.

The polymorphic behavior of lipid systems has been extensively investigated in lipid and food sciences, leading to the development of thermodynamic models capable of predicting solid–liquid equilibria and phase-transition properties in complex fat mixtures.^19,20^ Despite their maturity, these predictive frameworks have rarely been applied to thermal energy storage materials, representing an untapped opportunity for the development of biobased PCMs. Rather than developing new models from scratch, TES research can adapt these frameworks to investigate emerging lipid-based materials.

Methodology plays a critical role in this context. While DSC is a powerful tool for detecting thermal events with precision, its use of milligram-scale samples may not fully capture the bulk crystallization kinetics seen in practical TES systems.^21^ As a result, a great number of previous studies provide limited insight into how vegetable oils behave under the larger sample volumes and slower cooling rates characteristic of real TES applications. In contrast, the T-history^22^ method evaluates the thermal behavior of larger PCM samples, providing data that are more representative of macroscopic phase-transition behavior. Combining T-history with methods from the food industry enables the prediction and control of the thermal performance of vegetable oil-based PCMs.

To date, the integration between bulk-scale thermal characterization with composition-based thermodynamic predictions to interpret the crystallization behavior of vegetable oils for TES applications remains underexplored. In this context, the present study examines the impact of chemical composition and cooling conditions on the thermodynamic properties of coconut oil (a vegetable oil PCM candidate) during solidification. It offers valuable applications and validation of established composition-based thermodynamic predictions into TES analysis. By combining lipid-based prediction models with experimental measurements at an engineering scale, lipids can be repurposed to design and characterize novel PCMs. Furthermore, it enables investigation of heat transfer during the solidification process. Complementary to this, chemical characterization allows the prediction of the individual TAG contributions to the sample’s melting properties. Combining these predictions with experimental data enables the assessment of the significance of TAGs in the material’s overall phase transition behavior. It also allows identifying the temperature ranges in which the material is most effective.

## 2 Methodology

### 2.1 Coconut Oil Chemical Characterization

Two commercial coconut oil samples were purchased from local markets. The samples were used as provided, not requiring further preparation steps. The differences observed between the samples reflect the natural variability of vegetable oils, which depends on factors such as plant origin, cultivation conditions, and industrial processing, and are therefore inherent to commercially available products.^23,24^

The chemical characterization of the samples was performed using chromatography analysis, following the AOCS official methods Ce1-62 and Ce2-66.^25^ The gas chromatography (GC) analysis was carried out on a gas chromatograph (Shimadzu, model GC 2010 AF, Japan) with a flame ionization detector and an automatic injector (Shimadzu, model AOC 20i, Japan). A highly polar capillary column of bis-cyano propyl polysiloxane with 100 m of length, 0.25 mm of internal diameter, and 0.20 μm of film thickness was used (SP—2560, Supelco, USA), under the following chromatographic conditions:^24^ helium as carrier gas (with a linear speed of 19.5 cm/s); injector temperature 250 °C, column temperature 140 °C (for 5 min), 140 to 240 °C (at a rate of 4 °C/min), 240 °C (for 15 min); detector temperature of 260 °C, 100:1 split and injection volume of 1.0 μL. Fatty acid methyl esters were identified by comparison with the retention times of standards (Supelco 37 component FAME mix, USA). Quantification was performed based on the area ratios of each fatty acid to the area of the internal standard (methyl tridecanoate, >97% purity, Sigma-Aldrich, Bellefonte, USA, CAS 1731-88-0), using correction factors for the response of the flame ionization detector and the conversion of methyl esters into fatty acids.^26^

### 2.2 Triacylglycerol Composition Estimation

The fatty acid profile results from chromatographic analysis were used in a statistical method to predict the triacylglycerol composition of each sample.^27^ This method has been widely applied for TAG estimation and thermodynamic modeling of various fatty systems, with its detailed methodology described by Gonçalves et al.^28–30^ The triacylglycerol composition was estimated using a statistical model that requires, alongside fatty acid composition, the percentage of trisaturated TAG, which are triacylglycerols composed entirely of saturated fatty acids. In the current work, the trisaturated percentage was set to 92% for both samples, based on values reported.^31^

### 2.3 Thermal Properties Estimation

Melting temperatures and enthalpies of each triacylglycerol were estimated using a group contribution method initially proposed by Wesdorp.^32^ This method is commonly applied for estimating solid fat content (SFC) in complex lipid systems.^33–35^ The specific implementation, which employs the Wesdorp model with revised parameters proposed by Moorthy et al.^36^ was used in this study. The group contribution method accounts for the three possible fatty acid positions (Sn_1_, Sn_2_, Sn_3_) within the triacylglycerol molecule (Figure 1), as these positions impact thermodynamic and crystallization properties.^37^ For a detailed description of the method and its equations, refer to the cited literature.^36,38^

**Figure 1:**
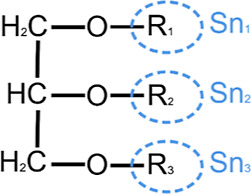
Triacylglycerol molecular structure. Positions Sn_1_, Sn_2_, and Sn_3_ represent the locations where fatty acids (R radicals) can be esterified.

The group contribution method begins by identifying chain length difference parameters (*x* and *y*). These parameters represent the carbon number disparities between the fatty acid on position Sn_2_ and those on position Sn_1_/Sn_3_ according to eqs 1 and 2

1 $$x=n_{2}-\min\left(n_{1},n_{3}\right)$$

2 $$y=\max\left(n_{1},n_{3}\right)-n_{2}$$

where *n*_1_, *n*_2_ and *n*_3_ the carbon number of fatty acids in positions Sn_1_, Sn_2_, and Sn_3_, respectively. The enthalpy and entropy of fusion of saturated compounds are calculated with eqs 3 and 4

3 $$\Delta H^{sat}=h_{n}+h_{0}+h_{xy}f_{xy}+h_{odd}f_{odd}$$

4 $$T_{m}^{sat}=T_{\infty }\left(1+\frac{A_{s}}{n}-\frac{A_{s}B_{s}}{n^{2}}\right)$$

where *h* is an enthalpy contribution, *n* is the total number of carbons from the fatty acid derivatives (*n* = *n*_1_ + *n*_2_ + *n*_3_), *h*_0_ is a contribution due to the glycerol headgroup, *h*_*xy*_ is a contribution due to the difference in chain length between fatty acid chains, *h*_odd_ is the contribution due to odd number of total carbons in the fatty acid derivatives, *f*_odd_ is an indicator function (1 if all fatty acid chains are even-numbered; 0 otherwise), *B*_s_, and *f*_*xy*_ are functions defined by eqs 5–7

5 $$f_{xy}=2-\exp\left[-{\left(\frac{x-x_{0}}{k_{x}}\right)}^{2}\right]-\exp\left[-{\left(\frac{y}{k_{y}}\right)}^{2}\right]$$

6 $$A_{s}=A_{0}+A_{odd}f_{odd}+A_{x}x+A_{x^{2}}x^{2}+A_{xy}xy+A_{y}y+A_{y^{2}}y$$

7 $$B_{s}=B_{0}+B_{odd}f_{odd}+B_{x}x+B_{x^{2}}x^{2}+B_{xy}xy+B_{y}y+B_{y^{2}}y$$

where *x*_0_, *k*_*x*_, *k*_*y*_, *A*_0_, *A*_odd_, *A*_*x*_, *A*_*x*_2__, *A*_*xy*_, *A*_*y*_, *A*_*y*_2__, *B*_0_, *B*_odd_, *B*_*x*_, *B*_*x*_2__, *B*_*xy*_, *B*_*y*_, and *B*_*y*_2__ are fitted parameters, whose values are available at Moorthy et al.^36^

For describing the properties of unsaturated compounds, it is necessary to account for the number of oleic (*n*_O_), elaidic (*n*_E_), linoleic (*n*_Li_), and linolenic (*n*_Lo_) chains in the triacylglycerol. If multiple unsaturated chains are present, the number of pairs of unsaturated fatty acid derivatives is also needed. Equations 8 and 9 are then proposed for estimating the melting enthalpy and temperature of unsaturated compounds.^36^

8 $$\Delta H^{unsat}=\Delta H_{f}^{sat}+h_{O}n_{O}+h_{E}n_{E}+h_{Li}n_{Li}$$

9 $$T_{m}^{unsat}=T_{\infty }\left(1+\frac{A_{u}}{n}-\frac{A_{u}B_{u}}{n^{2}}\right)$$

In eq 8, the enthalpy is first calculated as if the compound were saturated, using eq 3; then, the contributions of the unsaturated are considered. Parameters *h*_O_, *h*_E_, and *h*_L_ represent enthalpy contributions due to oleic, elaidic, and linoleic chains, respectively. Equation 9 is analogous to eq 4 except that *A*_s_ and *B*_s_ are substituted by *A*_u_ and B_u_ to incorporate the contribution of unsaturated compounds. *A*_u_ and B_u_ are calculated by eqs 10 and 11, respectively

10 $$A_{u}=A_{s}+A_{O}n_{O}+A_{E}n_{E}+A_{Li}n_{Li}+A_{Lo}n_{Lo}+A_{OO}n_{OO}+A_{EE}n_{EE}+A_{LiLi}n_{LiLi}+A_{LoLo}n_{LoLo}+A_{OLi}n_{OLi}+A_{OLo}n_{OLo}+A_{LiLo}n_{LiLo}$$

11 $$B_{u}=B_{s}+B_{O}n_{O}+B_{Li}n_{Li}+B_{Lo}n_{Lo}$$

where *n*_*ij*_ is the number of pairs between unsaturated fatty acids *i* and *j* (*i*,*j* ∈ {O,E,Li,Lo}), *A*_s_ and *B*_s_ are calculated with eqs 5 and 6; *A*_O_, *A*_E_, *A*_L_, *A*_LE_, *A*_OO_, *A*_EE_, *A*_LiLi_, *A*_LoLo_, *A*_OLi_, *A*_OLo_, *A*_LiLo_, *B*_O_, *B*_Li_, *B*_Lo_ are fitted parameters, whose values are available at Moorthy et al.^36^

### 2.4 Prediction of Bulk Properties

To move from a component-level description to a quantitative reconstruction of the bulk behavior, the thermodynamic contributions of individual TAGs were combined into a mixture-level formulation for solid fraction and enthalpy as functions of temperature. The solid fraction and bulk enthalpy of the samples were calculated as functions of temperature based on the predicted thermodynamic properties of the constituent TAGs, as expressed in eqs 12 and 13

12 $$SFC\left(T\right)=1-\sum_{i}z_{i}\theta_{i}\left(T\right)$$

13 $$H\left(T\right)=\sum_{i}y_{i}\Delta H_{f,i}^{p}\theta_{i}\left(T\right)$$

where SFC­(*T*) represents the solid fraction of the sample at a given temperature T, *z*_*i*_ is the molar fraction of TAG *i*, *y*_*i*_ is the molar fraction of TAG *i* in the liquid phase, and Δ*H*_f,*i*_^p^is the phase transition enthalpy of TAG*i* in polymorphic configuration *p*. θ_*i*_(*T*), is used to describe the temperature-dependent phase state of each TAG, taking the value 1 when TAG*i* is in the liquid phase and 0 otherwise.

This formulation enables the reconstruction of both the solid fraction evolution and the bulk enthalpy–temperature relationship as cumulative contributions of individual TAG phase transitions, providing a simplified yet physically meaningful approximation of the distributed phase transition behavior of multicomponent lipid systems.

### 2.5 T-History Experiments

#### 2.5.1 Experimental Setup

The T-history^22^ method was adapted in this work to provide precise control over the cooling conditions and enable bulk-scale evaluation of phase transitions. Unlike DSC, which is restricted to milligram-scale samples and relatively high cooling rates (∼1 °C·min^–1^), the adapted T-history setup allows the investigation of gram-scale samples (≈5 g) under controlled cooling rates down to 0.1 °C·min^–1^, bridging the gap between laboratory measurements and practical PCM conditions. These slow cooling conditions are essential for reproducing the gradual temperature variations observed in ambient environments, thereby enabling the observation of crystallization behavior, subcooling, and polymorphic transitions under TES-relevant conditions.

In this modified configuration, water was used as the cooling medium, with its temperature controlled by an ultrathermostatic bath (521TD, Ethik, Brazil), providing a homogeneous and highly reproducible thermal environment. Before cooling, the sample and reference were heated to an initial temperature (*T*_0_) of 40 °C, set 20 °C above their respective melting points, and held for 60 min to ensure complete melting and structural relaxation, thereby eliminating memory effects that influence crystallization.

Figure 2 compares the original and modified T-history setups. In the conventional configuration, the sample and a reference material with known thermophysical properties are heated to an initial temperature and then allowed to cool naturally under ambient conditions (Figure 2a). In the modified setup adopted in this work, cooling is instead imposed by a temperature-controlled bath following a linear ramp from the initial temperature (Figure 2b). The programmed cooling rate is selected to maintain thermal equilibrium in the absence of phase change, whereas deviations between the sample and bath temperature profiles reveal the onset of phase transitions and the extent of subcooling.

**Figure 2:**
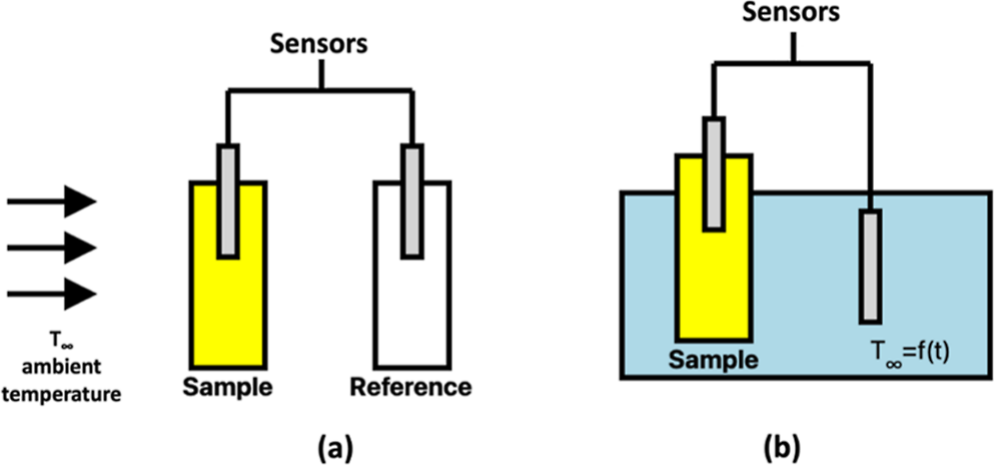
Schematic representation of the T-history configurations: (a) conventional setup, in which the sample and reference cool naturally under ambient conditions; and (b) modified setup, where cooling is imposed by a temperature-controlled water bath following a programmed thermal profile.

#### 2.5.2 Validation

To ensure the reliability of the adopted setup, the modified T-history setup was validated using *n*-eicosane (99% purity, Sigma-Aldrich, USA, CAS 112-95-8), a paraffin wax with well-characterized phase-transition properties. Validation was performed by comparing its cooling behavior with that obtained using a conventional T-history setup operating under ambient-air cooling (Figure 2). In both experimental configurations, DS18B20 sensors (±0.05 °C, thermal response time <750 ms) were used to record the temperature.

#### 2.5.3 Experiments

Following validation, the modified T-history setup was applied to study coconut oil under various cooling rates. Two coconut oil samples were analyzed. Approximately 5 g of sample was heated to 40 °C, ensuring they were completely melted, as the reported melting temperature of coconut oil ranges from 22 to 26 °C.^11^ This procedure is essential to eliminate any memory effects in the samples. The samples were kept at this temperature for 1 h. Then, the samples were cooled to 5 °C in different time periods ranging from 2 to 6 h. The stability of the thermal properties was investigated by subjecting a coconut oil sample to 18 of cooling and heating. In these cycles, the thermal bath was programmed to decrease linearly from 40 to 5 °C over 3 h, thereby cooling the sample under controlled conditions. The complete set of thermal histories evaluated in this study, including validation experiments and controlled cooling cycles for both coconut oil samples, is summarized in Table 1.

**Table 1:** Experimental Conditions for T-History Measurements of *n*-Eicosane (Validation) and Coconut Oil bioPCM Samples

substances/experiments	initial temperature (°C)	final temperature (°C)	cooling period (h)
Validation experiments			
*n*-eicosane—setup A	70	28	—
*n*-eicosane—setup B	70	5	6
Coconut oil bioPCMs			
Sample 1	40	5	2, 3, 4, 5, 6
Sample 2	40	5	2, 3, 4, 5, 6

## 3 Results and Discussion

The sequence of steps followed in this work is illustrated in Figure 3. First, the fatty acid composition of the coconut oil samples is quantified. Next, the corresponding TAG distribution is estimated, followed by the calculation of thermodynamic properties for each TAG. These properties are then used to reconstruct the bulk thermodynamic behavior of the samples. Finally, the predicted results are compared with experimental data.

**Figure 3:**
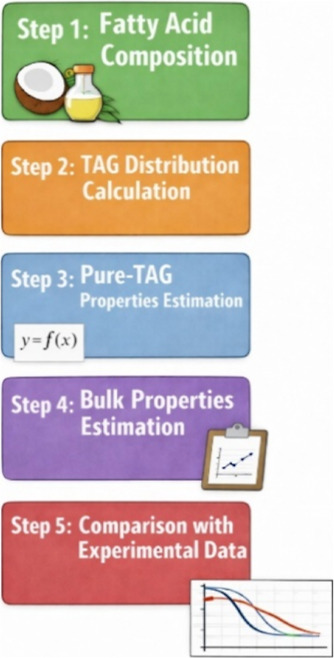
Schematic overview of the sequence of steps performed in this work.

### 3.1 Coconut Oil Composition Investigation

Table 2 reports the fatty acid composition for two coconut oil samples studied. The analysis of coconut oil composition is crucial for establishing the samples’ characteristics at the point of analysis and for interpreting of the variations in thermal behavior between samples in this study and those reported in the literature.^12^ Although individual fatty acids differ only slightly between samples,^39^ their grouped distributions reveal clear compositional trends. Sample 1 contains a higher proportion of medium-chain fatty acids (Cp, C, L), accounting for 63.7% compared to 52.7% in Sample 2, consistent with lower melting temperatures. In contrast, Sample 2 is enriched in long-chain saturated fatty acids (M and P), increasing from 24.7% in Sample 1 to 30.9%, as well as in unsaturated species (O and Li), from 8.4% to 13.6%. These compositional differences can influence melting temperature, crystallization kinetics, and polymorphic behavior, and are expected to impact the resulting triacylglycerol distribution, as discussed below.

**Table 2:** Fatty Acid Composition (Mass Basis) in Coconut Oil Samples, Sample 1 and Sample 2, Determined by Gas Chromatography

fatty acid	trivial name (abbreviation)	sample 1	Sample 2
C8:0	Caprylic (Cp)	8.3 ± 0.1	5.53 ± 0.02
C10:0	Capric (C)	6.4 ± 0.2	4.45 ± 0.08
C12:0	Lauric (L)	49 ± 2	42.7 ± 0.8
C14:0	Myristic (M)	16.8 ± 0.2	19.3 ± 0.2
C16:0	Palmitic (P)	7.9 ± 0.4	11.63 ± 0.08
C18:0	Stearic (S)	3.1 ± 0.2	2.88 ± 0.02
C18:1	Oleic (O)	6.1 ± 0.6	10.0 ± 0.3
C18:2	Linoleic (Li)	2.3 ± 0.2	3.6 ± 0.8

While fatty acid analysis provides a convenient analytical description, the thermal properties of vegetable oils are governed by their TAG composition. Direct TAG quantification is experimentally demanding; therefore, the TAG distribution was inferred from the fatty acid profiles using the Antoniosi method,^27^ a widely applied and validated statistical approach.^34,40–42^ In this notation, each letter denotes the fatty acid esterified to the glycerol backbone; for example, LLP corresponds to a TAG composed of two lauric acids and one palmitic acid.

Figure 4 presents the 21 major TAG species (above 1 mol %, normalized to this cutoff). LLL, LLM, and LLCp dominate Sample 1, whereas Sample 2 contains predominantly LLM, LLL, and LLP. Given that the thermophysical properties of vegetable oils arise from the collective behavior of their constituent TAGs, the compositional differences identified here provide a mechanistic explanation for the distinct thermal responses of the two samples. Based on the estimated TAG distributions, the average molar masses were calculated as 656.8 g·mol^–1^ for Sample 1 and 683.0 g·mol^–1^ for Sample 2, and are used in subsequent sections to express thermodynamic properties on a mass basis.

**Figure 4:**
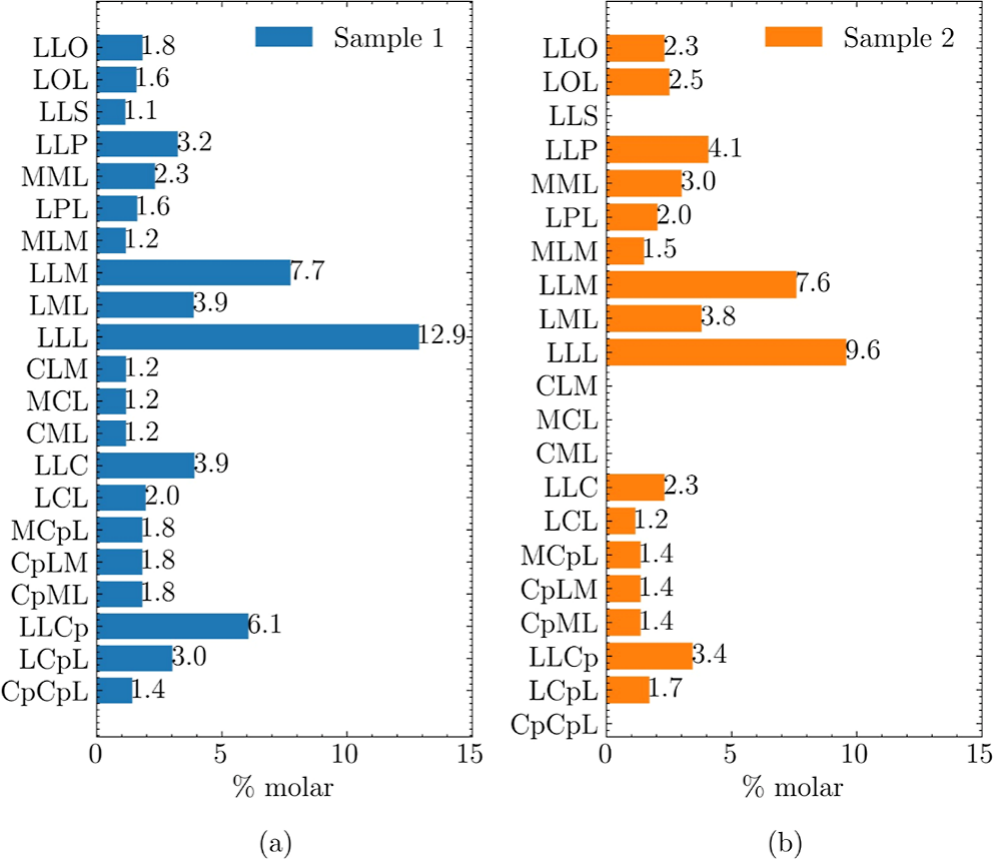
Triacylglycerol composition estimated for coconut oil samples based on the statistical analysis described in the Methodology section: (a) sample 1 and (b) sample 2. Abbreviations represent the fatty acids esterified to the glycerol backbone. For details on fatty acid abbreviations, refer to Table 2.

TAGs such as LLL, LLM, LLP, and LLCp differ in chain length, degree of saturation, and polymorphic stability; consequently, their relative proportions are expected to define not only the temperature range but also the distribution of latent heat during phase transition. To quantify these intrinsic differences, Table 3 compiles the predicted melting temperatures (*T*_m_) and enthalpies of fusion Δ*H*_m_ of the individual TAGs considered in the composition-based modeling, for the α, β′, and β polymorphic forms.

**Table 3:** Predicted Temperature and Enthalpy of Fusion of Individual Triacylglycerols Used for Composition-Based Modeling of Coconut Oil Phase-Transition Behavior^a^

	α		β′		β	
TAG (NC:unsaturations)	*T*_m_ (°C)	Δ*H*_m_ (kJ mol^–1^)	*T*_m_ (°C)	Δ*H*_m_ (kJ mol^–1^)	*T*_m_ (°C)	Δ*H*_m_ (kJ mol^–1^)
LLO (C45:1)	–5.8	37.5	15.7	63.5	10.3	92.5
CpCpL (C31:0)	–22.8	35.1	17.5	38.1	15.3	71.2
LOL (C45:1)	–9.8	40.6	17.6	79.4	15.4	94.6
LCpL (C35:0)	–6.4	44.4	19.2	68.3	34.5	86.5
LLC (C37:0)	4.1	57.0	20.0	82.1	35.2	102.9
LLCp (C35:0)	–8.0	42.7	23.9	59.9	28.3	72.3
CpLM (C37:0)	–1.3	44.3	24.7	67.3	32.9	77.1
LCL (C37:0)	5.6	54.3	26.7	76.2	39.8	101.7
CpML (C37:0)	–3.2	43.2	28.6	57.8	33.1	74.6
MCpL (C37:0)	2.5	47.3	30.6	63.7	33.4	85.7
LLL (C39:0)	14.5	64.2	33.1	88.0	44.6	121.3
CLM (C39:0)	9.5	57.4	34.4	83.1	37.1	100.1
MCL (C39:0)	10.1	57.2	34.6	71.6	38.9	100.9
CML (C39:0)	9.1	58.5	35.2	82.1	39.0	98.4
LML (C41:0)	19.8	70.3	38.5	109.8	48.1	126.9
LLM (C41:0)	17.9	67.1	38.9	83.4	43.9	120.6
MLM (C43:0)	25.5	70.5	42.3	99.3	51.7	125.0
LLP (C43:0)	21.1	67.5	43.0	84.4	44.1	117.9
MML (C43:0)	23.8	73.2	43.1	105.2	47.7	126.2
LPL (C43:0)	22.0	71.7	43.2	109.8	50.1	122.5
LLS (C45:0)	24.1	69.2	45.2	91.8	45.2	122.7

a Triacylglycerols are listed in ascending order of *T*_m_ for the β′ polymorph.

Overall, the melting temperature increases with the total number of carbon atoms in the TAG molecule, reflecting enhanced van der Waals interactions and packing efficiency in longer chains. However, the presence of unsaturation markedly disrupts this trend, leading to substantially lower melting temperatures, as illustrated by unsaturated TAGs such as LLO and LOL (C45:1), which melt at significantly lower temperatures than their saturated counterparts with comparable carbon numbers. Across all TAGs, the α form consistently exhibits the lowest melting temperatures and enthalpies, followed by the β′ and β forms, in line with their increasing thermodynamic stability.^17^ In contrast, the enthalpy of fusion does not scale linearly with melting temperature. For example, TAGs with similar *T*_m_ values in the β′ form may display markedly different Δ*H*_m_ values (e.g., LLCp versus LLC), indicating that latent heat is governed not only by transition temperature but also by molecular symmetry, packing efficiency, and polymorphic organization.

Table 4 first provides a global quantification of the transition enthalpy for each sample, assuming that all TAGs crystallize in a single polymorphic form. The enthalpy values for the α, β′, and β polymorphs are 54.0, 78.3, and 101.1 kJ·mol^–1^, respectively, when the temperature is sufficiently high for all TAGs to remain in the liquid phase. When converted to a mass basis, these values correspond to 82.2, 119.2, and 153.9 kJ·kg^–1^ for Sample 1, and 79.6, 118.6, and 151.2 kJ·kg^–1^ for Sample 2. These values are consistent with literature data for coconut oil, where DSC measurements typically report melting enthalpies in the range of 70–120 kJ·kg^–1^.^11^ Moreover, DSC studies on lipid systems show that the measured enthalpy varies with cooling rate, corroborating distinct crystallization pathways and polymorphic selection.^43^

**Table 4:** Predicted Molar Enthalpies of Phase Transition for Coconut Oil Samples, Assuming All TAGs Crystallize in the Same Polymorphic Form (α, β′, or β), and Comparing Calculations Based on the Complete Versus Major TAG Composition (21 TAGs)

	sample 1		sample 2	
conformation enthalpy (kJ mol^–1^)	all TAGs	major TAGs	all TAGs	major TAGs
α	54.0	36.4	54.4	34.2
β′	78.3	50.8	81.0	48.6
β	101.1	66.6	103.3	62.7

When the complete TAG distribution is considered, i.e., considering the full TAG distribution without applying the 1 mol % cutoff, the estimated molar enthalpy is reduced by approximately 30–35% compared to calculations including only the major components, regardless of polymorphic form. This reduction reflects the cumulative effect of minor TAGs, which decrease cooperative packing and lower the effective latent heat. The same trend is observed for both samples and across all polymorphs, indicating that the transition enthalpy is governed by the global TAG distribution rather than by individual TAG species.

These results highlight that, much like paraffin-based PCMs, the effective enthalpy and melting range of lipid-based bioPCMs can be engineered through controlled fractionation.^6^ The resulting composition- and polymorphism-based predictions provide a mechanistic framework that is directly validated against experimental T-history measurements in the following section.

While Table 4 captures the overall energetic scale of the phase transition, Figure 4 provides a temperature-resolved view of how this enthalpy is distributed among individual TAGs. Polymorphism primarily affects the temperature at which these contributions occur and their distribution across multiple transitions. As the crystalline form evolves from α to β′ and β, the main contributions shift to higher temperatures and become more localized, consistent with increased crystal stability. For both samples, the dominant enthalpy contributions arise from the same high–molar fraction TAGs, namely LLL and LLM, whose predicted transitions occur in comparable temperature ranges (14.5, 33.1, and 44.6 °C for LLL; 17.9, 38.9, and 43.9 °C for LLM across the α, β′, and β forms). Consequently, the main features of the thermal response are largely shared. Differences between samples arise primarily from the third most significant TAG contribution: in Sample 1, it is associated with LLCp (−8.0, 23.9, and 28.3 °C), whereas in Sample 2, it corresponds to LLP (21.1, 43.0, and 44.1 °C). This contrast reflects differences in TAG composition and polymorphic stability, leading to a shift in overall transition behavior and a lower effective phase-transition temperature for Sample 1 compared to Sample 2.

While Figure 5 provides a component-level description of the phase-transition behavior, the material’s practical performance depends on the collective response of all TAGs. Therefore, the next step is to integrate these contributions to estimate the system’s bulk thermodynamic properties as a function of temperature.

**Figure 5:**
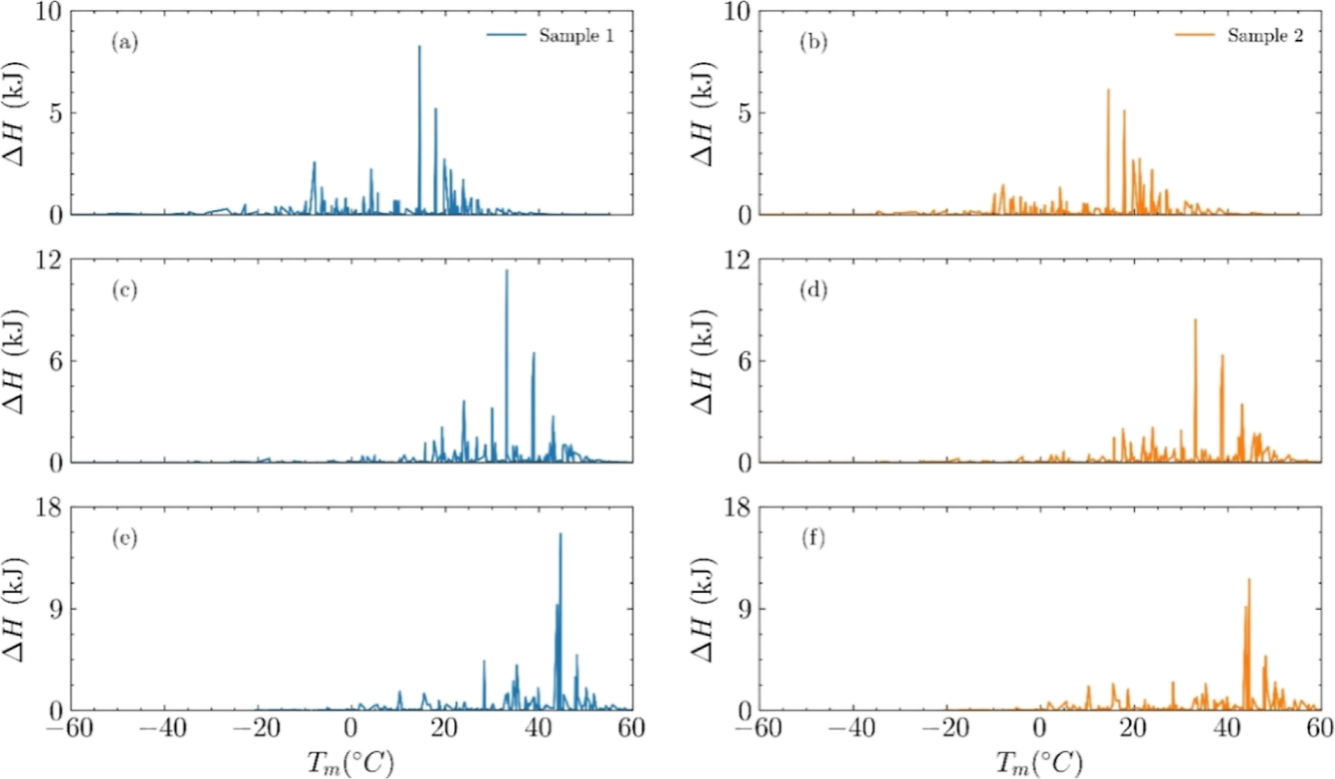
Estimated contribution of triacylglycerols to melting enthalpy in coconut oil, considering α, β′, and β conformations, for sample 1 (a,c,e) and sample 2 (b,d,f), respectively. TAG composition was estimated based on 25 and properties based on 34.

### 3.2 Bulk Properties Estimation

The bulk properties are directly related to the fraction of TAGs available to undergo phase transition. As temperature decreases, TAGs progressively crystallize, leading to continuous changes in both the solid fraction content (SFC) and the liquid-phase enthalpy. The evolution of SFC as a function of temperature is shown in Figure 6a. Rather than a sharp transition, SFC increases gradually over a temperature range, reflecting the sequential crystallization of TAGs with different thermodynamic stabilities. Differences between polymorphic forms indicate that crystallization occurs at distinct temperature intervals, with more stable forms contributing to earlier and more pronounced increases in SFC.

**Figure 6:**
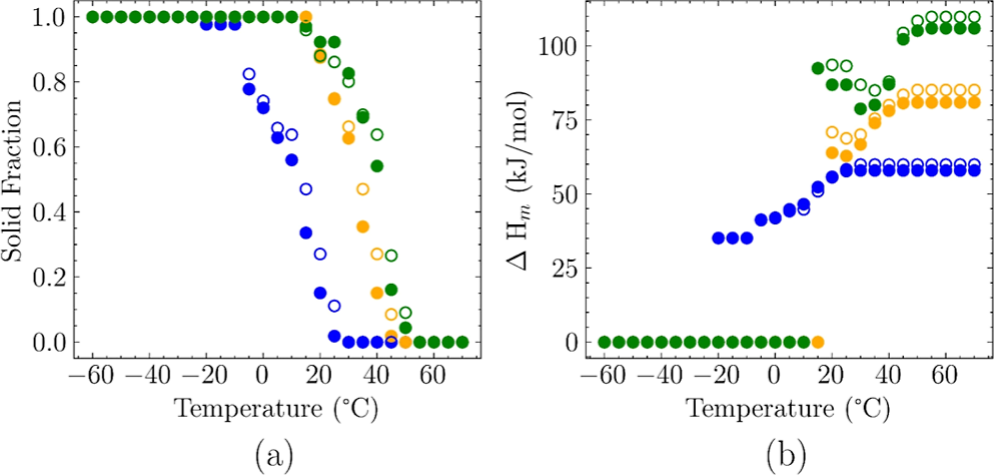
Temperature-dependent liquid-phase solid fraction content (SFC) (a) and enthalpy (b) for Sample 1 and Sample 2. Filled and open symbols correspond to Sample 1 and Sample 2, respectively. Colors indicate the polymorphic forms: α (blue), β′ (yellow), and β (green). The stepwise decrease in enthalpy reflects progressive TAG crystallization, which correlates with the increase in SFC and the distributed nature of the phase transition.

The corresponding evolution of the liquid-phase enthalpy is presented in Figure 6b. For these predictions, the 21 TAGs listed in Table 3 were considered. The stepwise decrease in enthalpy reflects the progressive crystallization of individual TAGs, with each drop corresponding to the partial solidification of specific components of the mixture. Each plateau, therefore, represents a distinct liquid-phase composition. At higher temperatures, the system remains compositionally complex, comprising a larger number of TAG species. As the temperature decreases, TAGs sequentially crystallize, progressively simplifying the composition of the remaining liquid phase. At lower temperatures, the liquid phase becomes dominated by the most thermodynamically stable TAGs (CpCpL for the α polymorph, and LLO for the β′ and β polymorphs). These results indicate that the phase-transition behavior of coconut oil is intrinsically distributed over a temperature interval and strongly dependent on both composition and polymorphic selection. This behavior is consistent with the expected response under nonequilibrium cooling conditions, as captured by the T-history approach.

### 3.3 T-History Experiments

#### 3.3.1 Validation

With compositional analysis and thermodynamic predictions indicating that the two samples are likely to melt at different temperatures, the next step was to experimentally examine their phase-transition behavior. To do this, a modified T-history setup was used. Before applying this method to coconut oil, the apparatus was validated with *n*-eicosane, a reference material with well-defined melting properties. Validation involved comparing the cooling behavior from the traditional ambient-air T-history configuration to that from the controlled-cooling setup used in this study (Figure 2).

Figure 7a illustrates the typical cooling behavior of substances in a constant temperature environment, following the standard T-history methodology proposed by Zhang and Jiang.^22^ The sample first undergoes exponential temperature decay associated with sensible cooling, followed by a slight temperature rise marking the onset of subcooling, and then a plateau corresponding to the phase-change region. After solidification is complete, the temperature returns to exponential decay. The phase-change temperature was 36 °C, within ∼2% of the 36.85 °C reported in the NIST database,^44^ confirming the reliability of the measurement.

**Figure 7:**
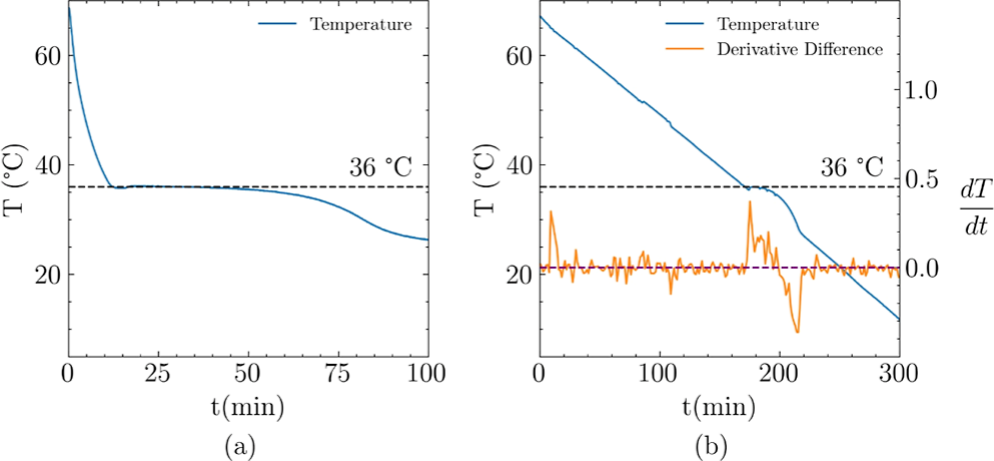
Cooling profile of *n*-eicosane under (a) ambient cooling (Setup A, standard T-history method) and (b) controlled cooling (Setup B). The black dashed line marks the crystallization temperature. In (b), the purple line represents the derivative of the temperature difference between the sample and the bath; a nonzero value indicates a temperature change due to the phase transition, rather than the heat source.

Understanding this phase-transition signature is particularly important for complex mixtures such as vegetable oils, in which multiple components can overlap in sensible- and latent-heat regimes (mushy zone).^45^ To address this complexity, the temperature derivative was used to identify the inflection point marking the end of the transition.^46^ During solidification, the derivative decreases gradually due to the overlap of sensible and latent heat effects and returns to a stable exponential trend once the transition is complete.

Figure 7b shows the corresponding behavior under controlled-cooling conditions (Setup B), in which the bath temperature decreases linearly with time. This configuration more closely represents the thermal fluctuations encountered in real building applications. Because both the bath and the sample experience the same imposed cooling rate, the difference between their temperature–time derivatives, ${\left(\frac{dT}{dt}\right)}_{sample}-{\left(\frac{dT}{dt}\right)}_{environment}$ , remains zero, except during *n*-eicosane’s phase transition near 36 °C, consistent with literature values.^47^ This result demonstrates that the modified setup reliably isolates the intrinsic phase-change signal from the imposed thermal boundary condition and provides a reproducible criterion for identifying phase transitions under dynamic cooling.

#### 3.3.2 Coconut Oil Cooling Behavior

With the T-history setup validated, the method was applied to the coconut oil samples to examine their phase-transition behavior under different cooling conditions. Cooling rates ranging from 0.3 to 0.1 °C·min^–1^ were selected to reproduce slow thermal variations, approaching the order of magnitude of ambient temperature fluctuations while remaining experimentally feasible within the T-history apparatus. Notably, daily temperature variations in building environments are typically on the order of ∼0.03 °C·min^–1^, placing them much closer to the slow cooling regime explored here (0.1–0.3 °C·min^–1^) than to the accelerated thermal ramps commonly imposed in DSC measurements.^48^ Although still faster than natural cooling, these rates represent a substantial improvement over DSC, which relies on cooling ramps orders of magnitude higher. Importantly, such slower gradients enable crystallization behavior to be assessed under conditions more representative of practical PCM operation, where nucleation and solidification are strongly influenced by gradual thermal changes.^48^

A clear dependence of the thermal response on the imposed cooling rate is observed for both samples (Figure 8). The cooling curves were analyzed using the same derivative-based criterion described previously, in which the phase-transition event is identified from deviations between the temperature–time derivatives of the sample and the imposed thermal environment. For Sample 1, fast cooling conditions (Case 1) lead to two well-resolved features in both the temperature and derivative profiles: a pronounced minimum associated with subcooling, followed by a distinct peak marking the onset of crystallization. As the cooling rate decreases, these features progressively converge, and under the slowest condition (Case 5) they nearly merge into a single transition, consistent with the reduced subcooling expected under milder thermal gradients.^37^

**Figure 8:**
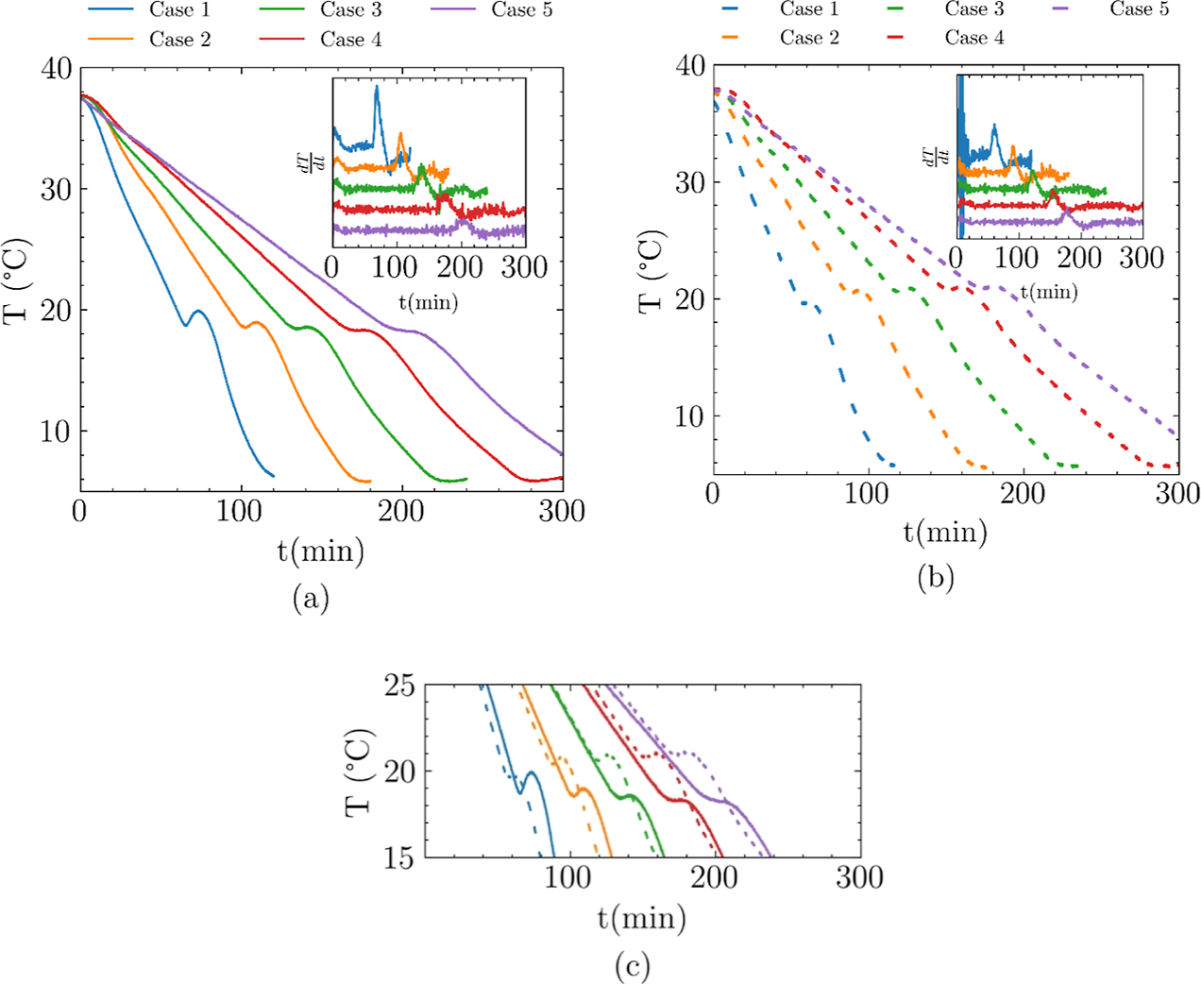
Cooling profile of two coconut oil samples: (a) Sample 1, (b) Sample 2, and (c) overlay comparison of Samples 1 and 2 under the same cooling conditions: Case 1 (blue)—2 h, Case 2 (orange)—3 h, Case 3 (green)—4 h, Case 4 (red)—5 h, and Case 5 (light purple)—6 h, for cooling from 40 to 5 °C.

In contrast, Sample 2 exhibits a different response to cooling rate. While the same derivative-based signatures are observed, both the subcooling temperature and the crystallization onset systematically shift to higher temperatures as cooling becomes slower. This behavior indicates an alternative crystallization pathway, likely associated with differences in composition and polymorphic selection under near-equilibrium conditions.

Figure 8c highlights that the two samples exhibit distinct cooling behaviors. Variations were also observed in key transition temperatures: the subcooling temperature *T*_sub_, the melting onset temperature *T*_m_, and the subcooling degree Δ*T* = *T*_m_ – *T*_sub_, as summarized in Table 5. Under the fastest cooling rate (Case 1), Sample 1 shows a markedly larger Δ*T* (1.2 °C) than Sample 2 (0.1 °C), indicating that a greater fraction of its released energy is spent overcoming metastability rather than being stored as latent heat. As the cooling rate decreases, Δ*T* for Sample 1 progressively diminishes, whereas Sample 2 maintains consistently low Δ*T* values across all conditions. These trends can be understood in terms of the crystallization kinetics of triacylglycerol systems. The arrangement of TAG molecules in the solid state is governed by factors such as the cooling rate and crystallization temperature, which control nucleation and crystal growth. Under slower and controlled cooling, molecules have sufficient time to organize into more stable lamellar and three-dimensional crystal structures, leading to reduced subcooling and more consistent thermal responses. In contrast, faster cooling promotes metastable polymorphic forms and kinetically limited crystallization, leading to greater subcooling and shifts in the observed transition temperatures.^37^

**Table 5:** Subcooling Temperature (*T*_sub_), Melting Temperature (*T*_m_), and Subcooling Degree (Δ*T*) for Coconut Oil Samples 1 and 2 under Different Cooling Conditions

	Sample 1			Sample 2		
Case	*T*_sub_ (°C)	*T*_m_ (°C)	degrees of Subcooling	*T*_sub_ (°C)	*T*_m_ (°C)	degrees of Subcooling
1	18.7	19.9	1.2	19.6	19.7	0.1
2	18.5	18.9	0.4	20.4	20.7	0.3
3	18.4	18.4	0.1	20.6	20.9	0.3
4	18.2	18.4	0.2	20.8	21.0	0.2
5	-	-	-	20.9	21.1	0.2

These differences in subcooling and melting behavior cannot be explained solely by cooling conditions; rather, they reflect the intrinsic molecular composition of the samples. Variations in triacylglycerol distribution and solid fat content can markedly shift crystallization pathways and transition temperatures: DSC analyses of virgin coconut oils from different regions show clear shifts in peak positions associated with differences in TAG composition and solid fat content;^49^ similar behavior is also observed for other vegetable oils. Similar effects have been reported for other vegetable oils, where even minor changes in TAG distribution significantly influence crystallization behavior.^50^

The higher transition temperature observed for Sample 2 under fast cooling conditions is consistent with these compositional and structural effects (Figure 5). Crystallization in fat-based systems is highly sensitive to both cooling rate and molecular arrangement, and TAG mixtures can follow different solidification pathways depending on their susceptibility to form metastable or stable polymorphs. Under nonisothermal conditions, faster cooling is known to favor the nucleation of the β′ polymorph, a metastable form generally associated with higher melting temperatures.^51^ This behavior has been documented for model TAG systems; for instance, Stapley et al.^52^ reported that PPP crystallizes predominantly as β′ under rapid cooling. Similar studies on coconut oil and simplified TAG mixtures confirm that polymorphic selection varies with both cooling rate and TAG composition.^53^

Triacylglycerols can adopt multiple crystalline arrangements, and their polymorphic transitions—typically classified into α, β′, and β forms—depend on both molecular structure and processing conditions,^37^ and the statistically estimated TAG compositions. Further evidence of compositional influence on crystallization comes from known TAG interactions. Each TAG species exhibits a characteristic induction time and activation Gibbs energy for nucleation; for example, SSS–OOO mixtures nucleate more slowly than PPP–OOO.^51^ Eutectic behavior can also shape crystallization: mixtures of SOS and LLL, for instance, form eutectics in their metastable forms, shifting and broadening crystallization peaks relative to pure components.^54^ Together, these molecular-level factors help explain the distinct thermal behaviors exhibited by the two coconut oil samples.

These compositional effects are reflected in the predicted TAG distributions shown in Figure 9. In particular, the distributions associated with the α polymorphic state (Figure 9b,f) are more concentrated and aligned within the temperature range where the phase transition occurs, indicating a stronger collective contribution of TAGs in this region. From an engineering standpoint, these effects imply that key PCM-relevant properties (such as transition temperature, subcooling magnitude, and effective latent-heat utilization) are not intrinsic constants of vegetable oils but system-level outcomes governed by TAG distribution and thermal history. This insight enables a rational design approach in which oil composition and cooling protocols are treated as engineering variables, allowing lipid-based PCMs to be tailored to application-specific temperature windows and operating conditions.

**Figure 9:**
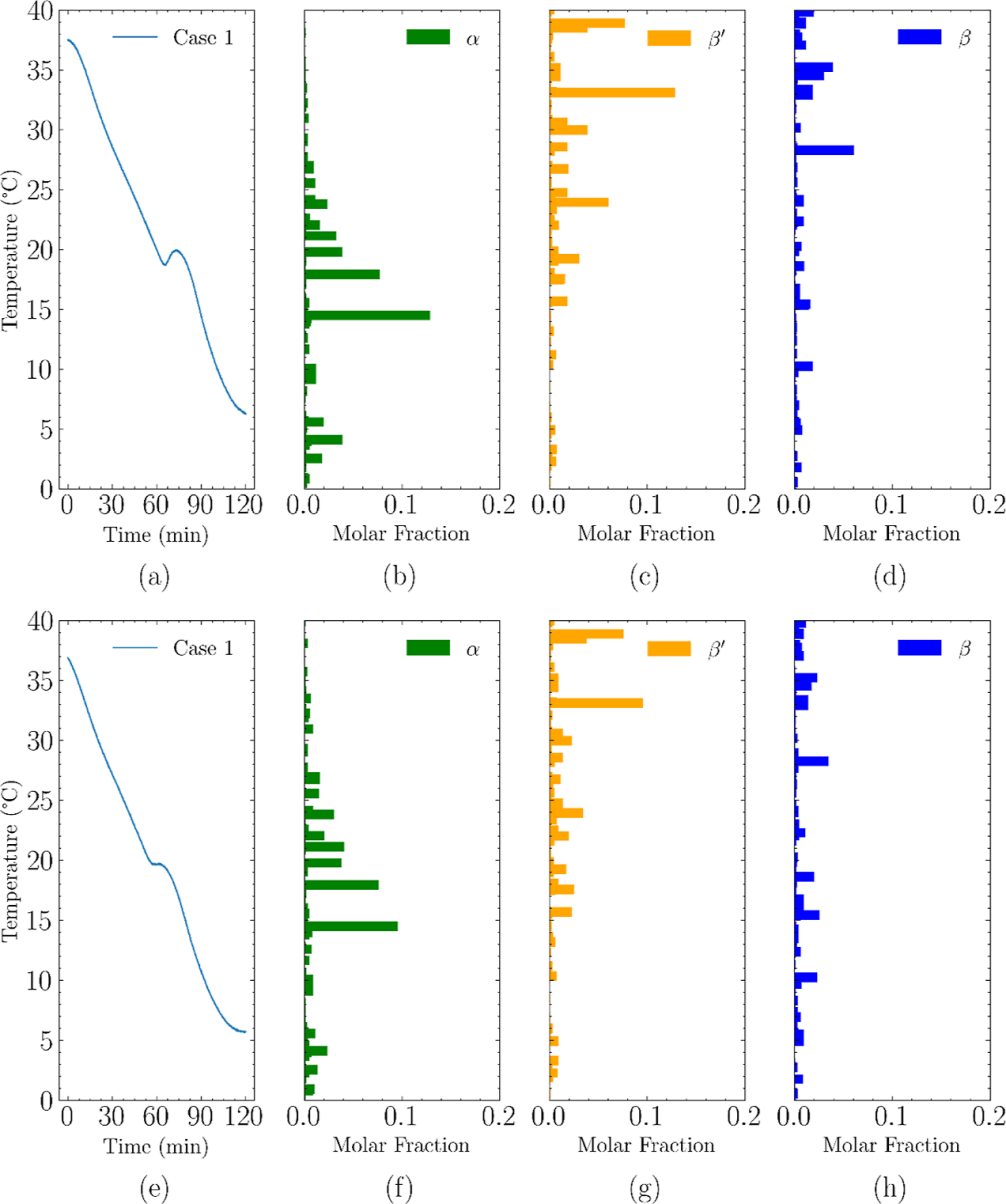
Comparison between T-history experimental results and predicted TAG contributions. Panels (a,e) show the cooling curves of Sample 1 and Sample 2, respectively. Panels (b–d,f–h) present the molar fraction distributions of TAGs as a function of predicted melting temperature for α, β′, and β polymorphic forms, respectively, for Sample 1 and Sample 2.

#### 3.3.3 Thermal Stability Assessment

Multiple heating–cooling cycles were performed to evaluate the stability of coconut oil under repeated thermal cycling. The sample was cooled from 40 to 5 °C over three hours and subjected to 18 consecutive cycles. Under these controlled conditions, the oil consistently exhibited a phase-transition temperature near 15 °C, with a mean subcooling of 15.4 ± 0.3 °C and an end-of-transition temperature of 13.7 ± 0.6 °C, values that exceed the sensor resolution (±0.05 °C) and therefore reflect true thermal behavior rather than measurement noise. The persistence of these values demonstrates that the observed subcooling behavior is intrinsic to the crystallization process and not associated with progressive chemical degradation. This agrees with prior reports showing that vegetable oils maintain their chemical and thermal integrity up to temperatures approaching 200 °C, beyond which degradation becomes significant.^55^ The temperature profile is presented in the Supporting Information (Figures S1–S3).

However, long-term applicability of lipid-based PCMs depends not only on chemical stability but also on the reproducibility of their phase-transition behavior. The literature presents mixed conclusions in this regard: Kahwaji and White^56^ observed small but noticeable differences in coconut oil after 5, 100, and 200 cycles, while other studies reported melting-temperature variations of up to 1.5 °C under repeated cycling.^57^ Such discrepancies likely arise from methodological differences, including insufficient residence time at high temperatures to erase crystallization memory, or cooling protocols that do not realistically impose the same thermal history in each cycle, both factors known to influence nucleation and crystallization pathways in fats.^51^

A further source of variability across studies is the absence of standardized testing methods and the frequent lack of detailed compositional information, particularly regarding fatty acid or TAG profiles. As emphasized by Ferrer et al.,^58^ establishing rigorous and harmonized procedures for assessing PCM stability is essential. The present results show that when both composition and cooling protocol are carefully controlled, coconut oil exhibits highly reproducible and stable phase-transition behavior, reinforcing its potential as a reliable biobased PCM for long-term thermal energy storage.

Our results highlight that coconut oil can indeed exhibit highly reproducible thermal behavior over multiple cycles, provided that two key factors are controlled: (i) the intrinsic composition of the oil and (ii) the imposed cooling conditions. Under well-defined conditions, the phase-transition characteristics are consistently reproduced with high fidelity, indicating that the observed behavior is governed by controlled crystallization pathways. These findings reinforce the potential of vegetable oils as robust, reliable phase-change materials for long-term thermal energy storage applications.

## 4 Conclusions

This study demonstrates that the phase-change behavior of coconut oil can be predicted, reproduced, and engineered when chemical composition and thermal history are rigorously controlled. By integrating bulk-scale T-history experiments with composition-based thermodynamic modeling, we establish a direct and quantitative link between triacylglycerol (TAG) distribution, polymorphic selection, and macroscopic phase-transition performance. The predictive methodologies employed show strong internal consistency and experimental agreement. Composition-based thermodynamic estimates captured both the transition temperature range and the magnitude of latent heat, yielding predicted molar enthalpies between ∼54 kJ·mol^–1^ (α form) and ∼100 kJ·mol^–1^ (β form) when the full TAG distribution was considered. These results confirm the suitability of lipid thermodynamic frameworks, long established in food science, for predicting the thermal behavior of biobased PCMs. The adapted T-history methodology proved to be a reliable and application-relevant experimental tool. Validation with *n*-eicosane, a reference PCM with well-established properties, showed excellent agreement with literature values, confirming the method’s ability to isolate intrinsic phase-change signals under controlled cooling. Unlike DSC, the modified T-history setup enables gram-scale measurements under slow cooling rates (0.1–0.3 °C·min^–1^), conditions far closer to those encountered in real thermal energy storage systems. Repeated thermal cycling further demonstrated excellent stability, with transition temperatures remaining within <5% deviation over 18 consecutive cycles, confirming that the observed behavior is intrinsic and not associated with chemical degradation or progressive structural drift.

Overall, coconut oil combines low subcooling, moderate transition temperatures, distributed latent heat, and high cycling stability, defining a high-quality phase-change response for low-temperature thermal energy storage. More broadly, this work shows that vegetable oils should not be treated as single-transition PCMs. Instead, their thermal performance can be rationally designed through composition control and crystallization management, analogous to paraffin fractionation strategies. The combined predictive–experimental framework validated here provides a robust pathway for engineering reliable lipid-based bio-PCMs with tunable thermophysical properties. These results further indicate that variability in reported behavior is not intrinsic to lipid-based systems but is largely governed by crystallization kinetics and polymorphic transitions, which can be effectively managed through controlled thermal protocols.

## Supplementary Material



## Notes

Cooling behavior of coconut oil submitted to 18 controlled cooling cycles, including 2D temperature profiles (Figure S1), 3D overlay of cooling curves (Figure S2), and enlarged view of the phase transition region between 90 and 140 min (Figure S3) (PDF)
