# Supplementary material for: Effects of Chemical Composition and Controlled Cooling on the Phase Transition in Coconut Oil Bio-Phase Change Materials
Source: ACS Omega. 2026 Jul 2;11(30):44788–800. doi: 10.1021/acsomega.6c01295 (PMC13449208; doi:10.1021/acsomega.6c01295)
Supplement: Supplementary file 1 [file ao6c01295_si_001.pdf]

Supporting Information

Effects of chemical composition and controlled cooling on the phase transition in coconut oil bio-Phase Change Materials

Matheus N. Guedes[a], Erislene S. Almeida[a], Christianne E. C. Rodrigues[b], Roberta F. Neumeister[a], Taygoara F. Oliveira[a], Antonio C.P. Brasil Junior.[a], Simone Monteiro[a*]

a. University of Brasília, Mechanical Sciences Graduate Program, Laboratory of Energy and Environment, Brasilia-DF, 70910-900, Brazil.

b. University of São Paulo (USP), Department of Food Engineering, Separation Engineering Laboratory, Pirassununga-SP, 13635-900, Brazil.

* Corresponding author E-mail: simonems@unb.br

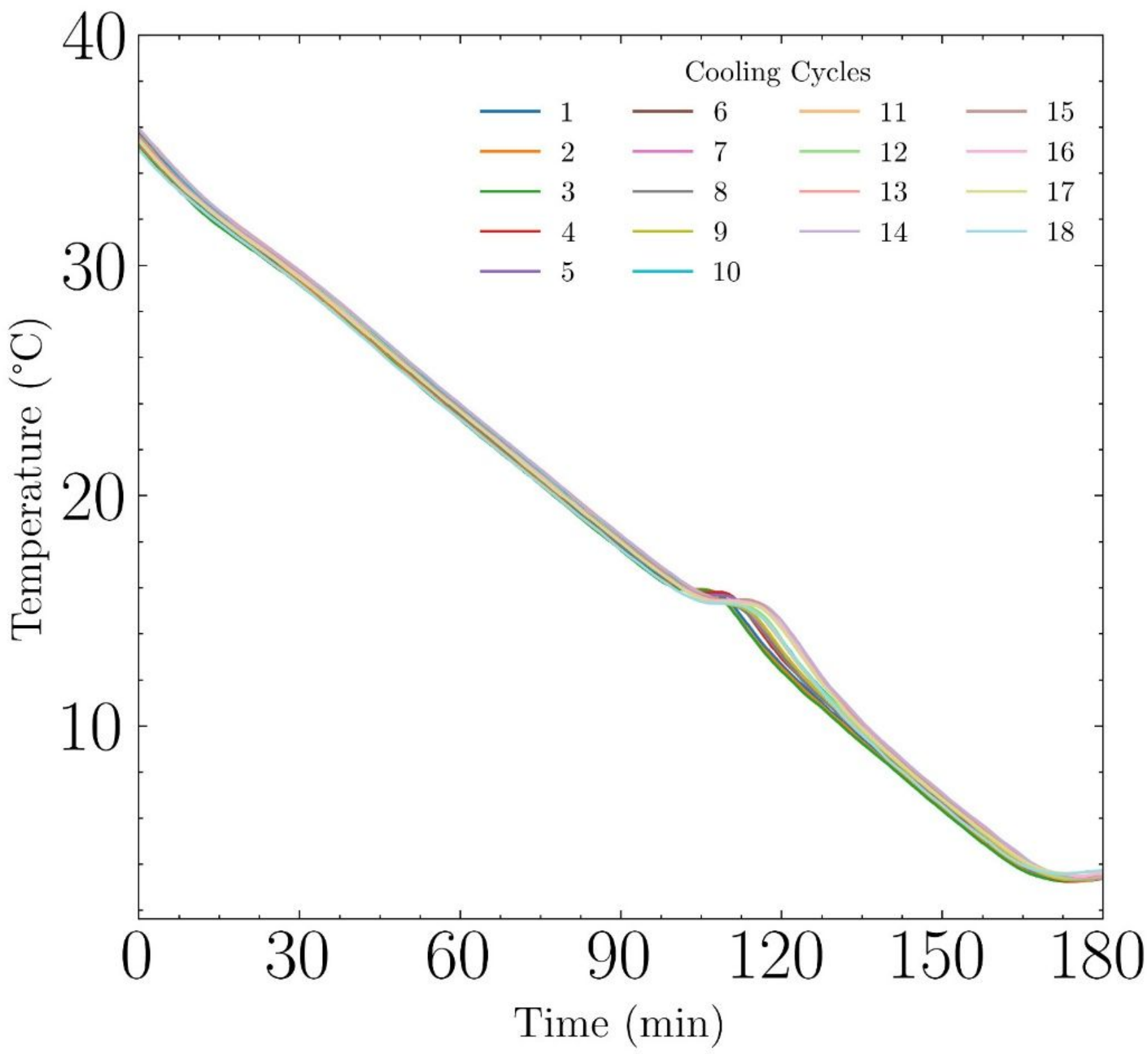

Figure S1: Cooling behavior of coconut submitted to 18 cooling cycles - a 2D representation of the temperature profile

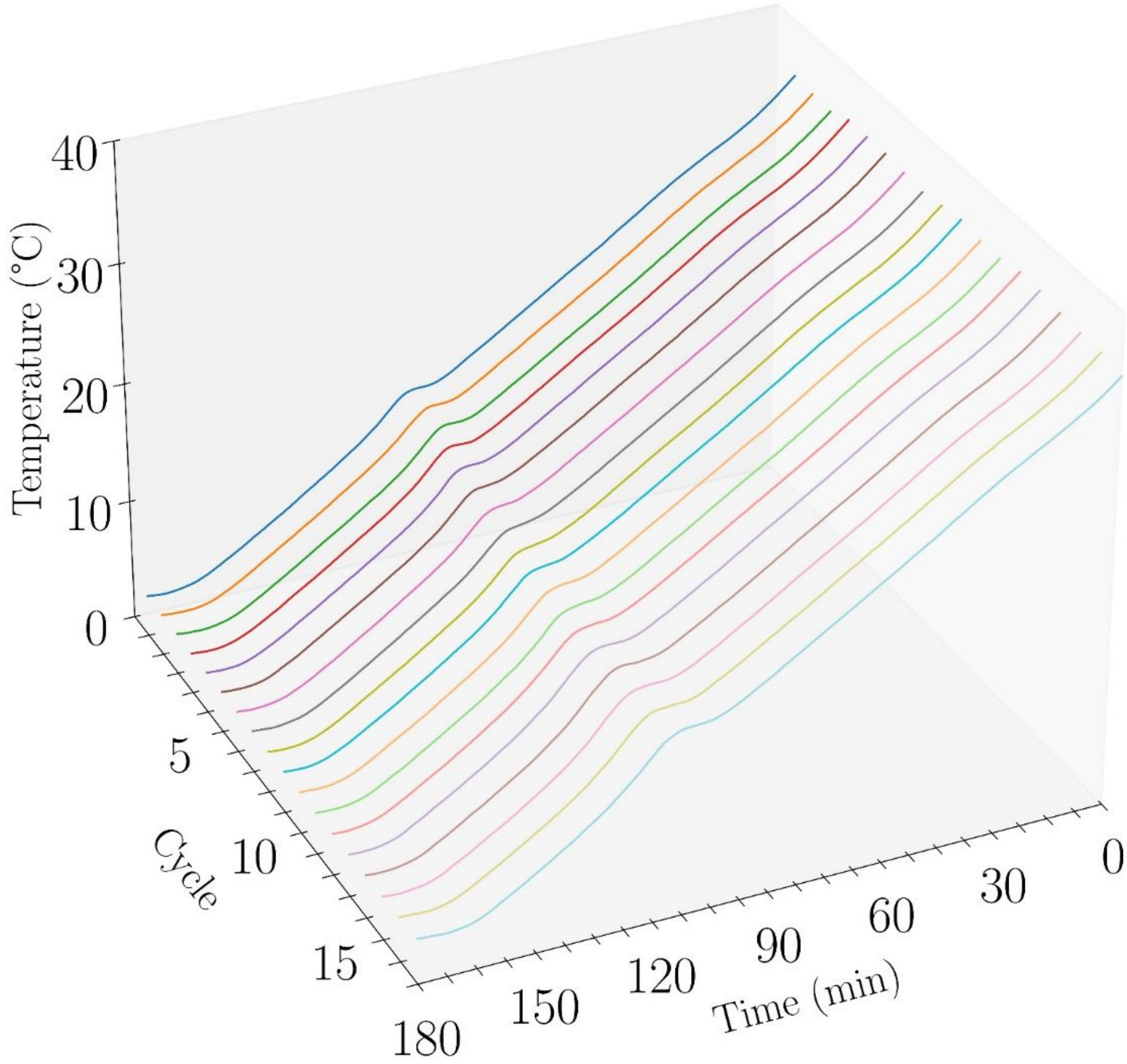

Figure S2: Cooling behavior of coconut submitted to 18 cooling cycles - an overlay of the curves in a 3D plot

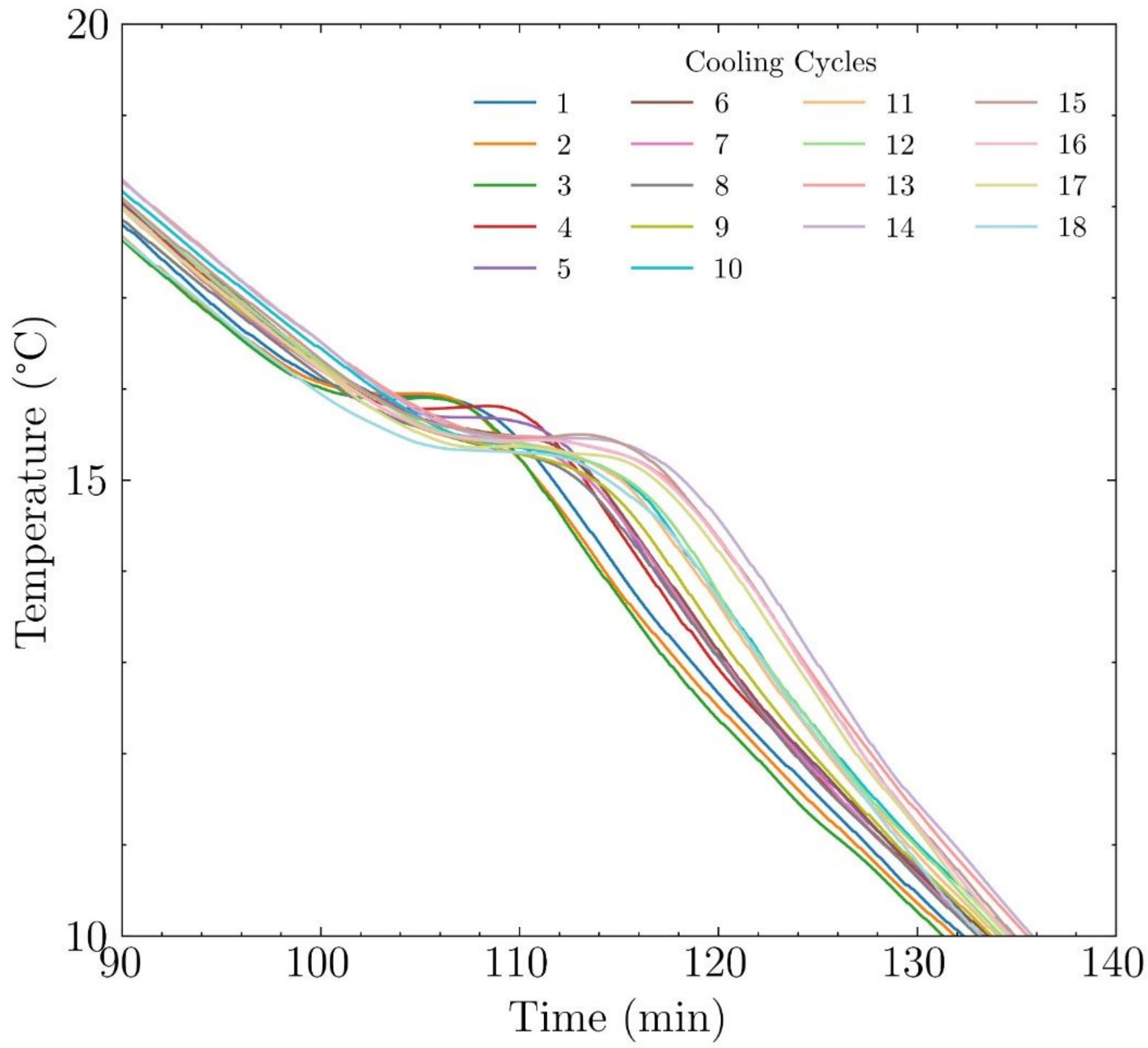

Figure S3: Cooling behavior of coconut submitted to 18 cooling cycles – zoom in the region between 90-140 min capturing the phase transition region.
